# Adropin’s Role in Energy Homeostasis and Metabolic Disorders

**DOI:** 10.3390/ijms23158318

**Published:** 2022-07-28

**Authors:** Ifrah Ismail Ali, Crystal D’Souza, Jaipaul Singh, Ernest Adeghate

**Affiliations:** 1 Department of Anatomy, College of Medicine & Health Sciences, United Arab Emirates University, Al Ain P.O. Box 17666, United Arab Emirates; 200540058@uaeu.ac.ae (I.I.A.); crystal.dz@uaeu.ac.ae (C.D.); 2 School of Natural Sciences, University of Central Lancashire, Preston PR1 2HE, UK; jsingh3@uclan.ac.uk; 3 Zayed Foundation, United Arab Emirates University, Al Ain P.O. Box 17666, United Arab Emirates

**Keywords:** *Enho* gene, adropin, carbohydrate metabolism, lipid metabolism, energy homeostasis

## Abstract

Adropin is a novel 76-amino acid-peptide that is expressed in different tissues and cells including the liver, pancreas, heart and vascular tissues, kidney, milk, serum, plasma and many parts of the brain. Adropin, encoded by the *Enho* gene, plays a crucial role in energy homeostasis. The literature review indicates that adropin alleviates the degree of insulin resistance by reducing endogenous hepatic glucose production. Adropin improves glucose metabolism by enhancing glucose utilization in mice, including the sensitization of insulin signaling pathways such as Akt phosphorylation and the activation of the glucose transporter 4 receptor. Several studies have also demonstrated that adropin improves cardiac function, cardiac efficiency and coronary blood flow in mice. Adropin can also reduce the levels of serum triglycerides, total cholesterol and low-density lipoprotein cholesterol. In contrast, it increases the level of high-density lipoprotein cholesterol, often referred to as the beneficial cholesterol. Adropin inhibits inflammation by reducing the tissue level of pro-inflammatory cytokines such as tumor necrosis factor alpha and interleukin-6. The protective effect of adropin on the vascular endothelium is through an increase in the expression of endothelial nitric oxide synthase. This article provides an overview of the existing literature about the role of adropin in different pathological conditions.

## 1. Introduction

Peptides are short linear chains of amino acids that are often stabilized by disulfide bonds. Peptides can be synthesized and used for different purposes and the sequence can also be modulated using various chemical and biological techniques [1,2,3,4,5,6,7,8]. There are therapeutic peptides, which are used to fight certain diseases such as cancer and diabetes mellitus and its complications [9,10,11]. 

Therapeutic peptides have several important advantages when compared to proteins and antibodies. They are smaller in size, easy to synthesize and have the ability to penetrate cell membranes. They also have high activity, specificity and affinity. An added benefit of using peptides as a therapeutic agent is that they do not accumulate in specific organs such as the kidney or liver, which can help to minimize their toxic side effects [12]. Therapeutic peptides show great potential in the treatment of many diseases [9,10,11,13]. One of these promising peptides is adropin. Adropin is a short peptide that consists of 76 amino acids. It was discovered by Kumar and colleagues in 2008. They demonstrated its role in glucose and lipid metabolism and energy homeostasis [14]. This review examines adropin peptide from different aspects, starting from the discovery and its biochemical structure to tissue localization. It also outlines the results of human and animal studies on the effects of adropin on physiological and biochemical parameters.

### 1.1. Discovery of Adropin

Adropin was discovered by Kumar et al. in 2008 [14], while studying the hypothalamic regulation of liver metabolism, and through conducting a microarray analysis of total gene expression in the liver of C57BL/6J mice that were deficient in melanocortin-3 receptor (*Mc3r ^−/−^*). Kumar and his team distinguished a novel transcript in the liver. This transcript encoded a short and highly conserved amino acid sequence (76 aa), which was downregulated in the obese mice. Further investigation revealed that the coding gene was associated with energy homeostasis and lipid metabolism, so it was named the Energy Homeostasis Associated (*Enho*) gene. The name “adropin” was derived from the initials of Latin names “aduro”, which means “to set fire to”, and “pinquis”, which means “fats” [14]. 

Adropin level has been shown to be related to nutrient intake. For example, the investigation of Kumar et al. showed that lean C57BL/6J mice that were fed a high-fat diet (HFD) expressed higher adropin in the liver, compared to the control group, while fasting lean C57BL/6J mice showed a diminished adropin level. Interestingly, another experiment in which diet-induced obesity (DIO) mice were used demonstrated that the level of liver *Enho* gene expression was reduced, compared to that of lean mice, explaining that the long-term intake of a high-fat diet disrupts the normal activity of adropin. This finding linked the *Enho* gene to metabolic disorders, such as obesity [14]. Further investigations showed that the blood level of adropin was markedly reduced in obese individuals when compared to those of normal subjects [15,16].

It has been reported that adropin is a secreted, as well as membrane-bound peptide. Previous reports show that adropin is secreted by HEK293 cells and C57BL/6J mice [14]. Other reports have indeed shown that adropin is secreted by brain tissue [16], while other investigators showed that the liver is a strong producer of adropin [17]. In contrast to these reports, there are reports that show that adropin is secreted by many types of tissues in the human body [18].

In contrast, a study that was conducted by Wong et al. showed that adropin was a membrane-bound peptide [19]. Initially, a bioinformatic analysis was used to make a prediction of the transmembrane topology of adropin. It was suggested that amino acids 1–9 of the N-terminal are cytoplasmic, while amino acids 9–30 are transmembrane-bound, and amino acids 30–76 are localized outside of the surface of the plasma membrane. Afterward, an immunohistochemical analysis showed that anti-adropin antibody co-localized with pan-cadherin antibody (membrane marker) using HeLa cells that were transfected with plasmid-expressing adropin. Moreover, it was demonstrated that adropin is expressed on the plasma membrane of C57BL/6N mice brain tissue and HEK293 cells. Despite all of these initial observations regarding the nature and location of adropin, most reports now point to the fact that adropin is indeed a secreted protein.

### 1.2. Enho Gene and Adropin Peptide Characteristics

**Figure 1:**
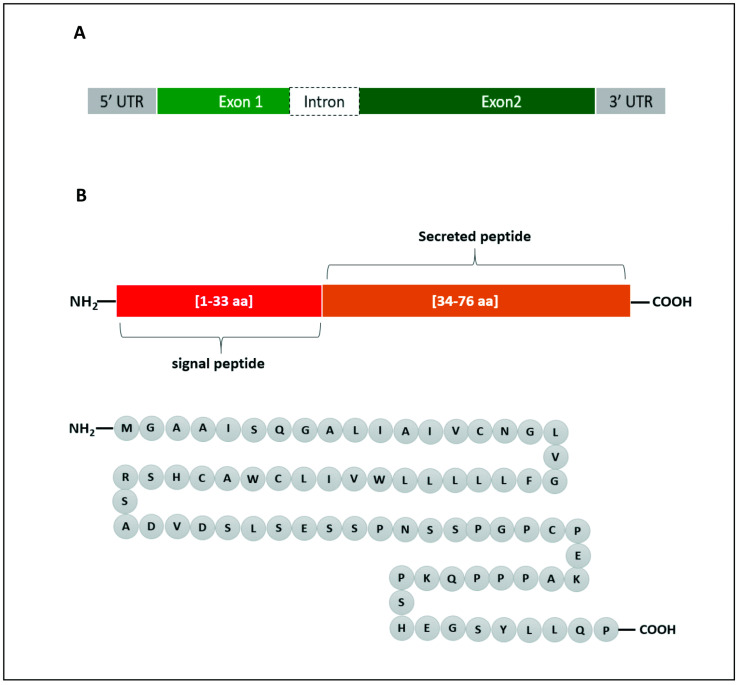
Graphical representation of *Enho* gene (**A**) and the encoded adropin (**B**). The gene consists of 2 exons and 1 intron. Adropin has a secretory sequence and a bioactive sequence. The full peptide is made up of 76 amino acids (aa).

In human, the *Enho* gene is in chromosome number 9 (9p13.3), position 34,521,043–34,522,990 and it has a size of 1948 bp. It is composed of two exons and one intron (Figure 1A). Adropin, which is encoded by the *Enho* gene, is 76 amino acids in length (MGAAISQGALIAIVCNGLVGFLLLLLWVILCWACHSRSADVDSLSESSPNSSPGPCPEKAPPPQKPSHEGSYLLQP) and has a molecular weight of 4.499 KDa (Figure 1B). The amino acid sequence is 100% conserved in human, mouse and rat. Adropin^1−33^ (amino acids 1–33) is a secretory signal peptide [14], and adropin^34−76^ (amino acids 34–76) is biologically active when it is administered to rats [20].

The literature has not stated a specific receptor upon which adropin exerts its biological activities. In the brain, Stein et al. showed that adropin acts on the orphan G protein-coupled receptor (GPR19) to inhibit water deprivation in rats [21], while in the liver, Kumar et al. demonstrated that *Enho* gene expression is regulated by Liver X receptor (LXR) [14]. LXR is a nuclear receptor, which also acts as a lipid and glucose sensor [22].

It was demonstrated that adropin stimulated angiogenesis, proliferation and migration of the human umbilical vein and coronary artery endothelial cells. It was also proposed that this novel peptide may exert its activities on vascular endothelial growth factor receptor-2 (VEGFR2) in endothelial cells [23]. 

## 2. Tissue Expression of Adropin

The early discovery of adropin demonstrated the expression of the peptide in the liver [14] and brain [19]; thereafter, the tissue distribution of adropin has been shown in various tissues and cells [18]. In the liver, the expression was detected in sinusoidal cells, while in the central nervous system (CNS), immunoreactivity of the peptide was present in the vascular area, pia matter, neuroglial cells, Purkinje cells, granular layer and neurons of the central nervous system of rat [24]. 

In the pancreas, adropin was detected in acinar cells [24] and in the capillaries of the islets of Langerhans [25] using immunohistochemical technique. Adropin immunoreactivity was observed in the capillaries of the renal glomeruli, peritubular interstitial and peritubular regions [26].

In the circulatory system, Lovren et al. showed that adropin was expressed in endothelial cells—specifically, in cultured human umbilical vein and coronary artery endothelial cells (ECs) [23]. Moreover, this study suggested a vascular effect for adropin through endothelial cells by enhancing capillary-like tube formation and exhibiting greater cell proliferation and migration. Furthermore, a histological analysis displayed the localization of adropin in the endocardium, myocardium and epicardium of rat heart [24]. Several other studies [27,28,29,30,31,32,33] have indeed linked endothelial function to adropin, thus confirming the probable presence in endothelial cells.

Furthermore, mRNA expression of the *Enho* gene has been detected in the lung tissue of C57BL/6J mice [34]. Interestingly, the same study reported genetic variations in the *Enho* gene after analyzing myeloperoxidase (MPO) and anti-neutrophil cytoplasm autoantibody (ANCA) in patients with vasculitis and healthy individuals [34].

It was demonstrated that biological fluids such as milk, serum, plasma and cheese whey milk-derived fluid of cow diary contain numerous amino acids and proteins including adropin, nesfatin-1, apelin-12, ghrelin and salusin peptides [35]. Additionally, human serum adropin was measured and correlated well with distinct diseases in several studies, especially coronary artery disease [35,36,37,38,39].

## 3. Adropin and Energy Homeostasis

The biological process of regulating energy inflow (food intake) and energy expenditure through biosynthetic reactions is identified as energy homeostasis. To maintain energy homeostasis, the amount of energy intake must be at equilibrium with the amount of energy that is expended. As the body intakes nutritional molecules such as carbohydrates, lipids and proteins, which are considered fuel, the required energy is utilized by the body and the excess is stored in a form of adipose tissue and held constant over a period of time [40].

The gastrointestinal tract (GI tract), pancreas and liver are known to provide hormonal signals to specific nuclei of the central nervous system that regulate the energy that is consumed and the energy that is utilized. These hormones include ghrelin; gastric leptin; secretin; glucagon-like peptide 1, secreted by the stomach and intestines; and insulin and glucagon, released from the endocrine pancreas. Adipose tissue is also a major source of energy-related signals—specifically, signals that reflect lipogenesis, storage and lipolysis. These signals send feedback to the region of the central nervous system that controls energy balance [41].

The brain, specifically the medial and lateral nuclei of the hypothalamus, plays a major role in energy homeostasis, with the help of hormonal, as well as nutritional signals. Food intake is controlled by hunger and regulated by the hypothalamus. There are several models related to food intake and energy homeostasis. The lipostatic model says that adipose tissue produces signals such as leptin, which is proportionate to the fat mass and acts on the hypothalamus to decrease food intake and increase energy output, while the GI tract-peptide model, hypothesizes that when the food enters the gastrointestinal tract, the release of cholecystokinin and glucagon hormone are induced. These hormones have receptors in the hypothalamus, which produce the feeling of fullness. Another model of energy balancing and food intake involves glucose utilization in brain neurons [40].

During feeding and fasting cycles, carbohydrates and fatty acids are the two primary substrates in oxidative metabolism to keep energy balanced. Several studies have reported the role of the hormonal peptide, adropin, in regulating substrate oxidation preferences. Initially, when adropin was discovered, its biological role was linked to glucose homeostasis and lipid metabolism [14]. Kumar et al., however, showed that liver *Enho* expression is regulated by the leptin and melanocortin receptor, as obese leptin knockout mice (Lep^ob^/Lep^ob^) and melanocortin 3 receptor knockout mice (Mc3r^−/−^) exhibited downregulation in *Enho* expression [14]. Melanocortin 3 receptor (Mc3r) is a member of the melanocortin receptors family, highly expressed in brain, as well as other tissues such as liver. The Mc3 receptor is involved in energy homeostasis, and the absence of the *Mc3r* gene causes increased adiposity in tissues [42]. Leptin is encoded by the *ob* gene and is secreted into the blood circulation by adipose cells. It also acts on hypothalamic receptors to inhibit feeding and initiates increased thermogenesis. It is an important regulator of energy homeostasis [43]. Another significant finding that was reported by Kumar et al., while investigating how nutritional status affects liver *Enho* mRNA expression, was that a high-fat diet increased *Enho* mRNA expression in lean C57BL/6J mice. However, introducing a high-fat diet to diet-induced obesity (DIO) C57BL/6J mice in a period of 3 months was associated with reduced liver *Enho* mRNA expression, compared to lean controls. This suggests that a chronic obese state caused metabolic disorder, which consequently disturbed adropin expression.

Additionally, adropin metabolism is not only associated with lipid, but also with carbohydrate metabolism and energy status. It is worth mentioning that adropin-overexpressing transgenic C57BL/6J mice that were fed a high-fat diet showed improvement in glucose homeostasis, as well as delayed development of obesity. Normally, a high-fat diet leads to obesity and disruption in glucose homeostasis [14,44].

In another study, elevated fasting glucose level in DIO mice was controlled and regulated by administrating an intraperitoneal injection of adropin. It was found that endogenous hepatic glucose production was reduced in adropin-treated obese mice [16,18,45,46,47,48]. 

## 4. The Role of Adropin in Health and Disease

The levels of adropin in blood circulation have been proposed to direct the metabolic state in skeletal muscle by influencing fuel selection preference towards glucose oxidation in the fed state [20]. Studies have shown that adropin regulates the expression of hepatic lipogenic genes and the PPARγ receptor (peroxisome proliferator-activated receptor gamma), the major regulator of lipogenesis [14]. Moreover, adropin regulates angiogenesis, increases blood flow, boosts capillary density and has a protective role for endothelial cells [23]. Apparently, the tissue level of adropin varies in several physiological and biological conditions such as multiple sclerosis [49], COVID-19 [50], gestational diabetes [51] obstructive sleep apnea [52], rheumatoid arthritis [53], coronary artery ectasia [54], acute mesenteric ischemia [55] and diabetic nephropathy [56].

### 4.1. Diabetes Mellitus

When adropin was first discovered, most of the attention was given to its role in lipid and carbohydrate metabolism and insulin resistance. Interestingly, some studies show that adropin deficiency plays a role in the development and progression of chronic diseases, such as diabetes mellitus. Zang et al. reported that serum concentrations of adropin were significantly decreased in Chinese type 2 diabetic patients, compared to control subjects [57]. Other studies confirmed this finding by reporting the downregulation in circulating adropin in adults with type 2 diabetes mellitus [58], liver disease [59] and children with type 1 diabetes mellitus [60]. In contrast, it was elucidated that higher insulin resistance and higher fasting plasma glucose positively correlated with serum adropin levels in patients with type 2 diabetes mellitus [61].

Additionally, low adropin levels have been shown to correlate with a risk of developing diabetic complications such as diabetic retinopathy [62], diabetic nephropathy [63] and gestational diabetes mellitus [64].

As it was reported that serum adropin levels vary between diabetic and normal subjects, several investigations tried to understand the mechanisms underlying these variations. For instance, it was demonstrated that hyperglycemia was associated with increased adropin expression, as well as the signal transducer and activator of transcription 3 (STAT3) activation in the liver of streptozotocin-induced diabetic rats. The mechanism underlying the elevation of adropin levels and *Enho* gene expression in the diabetic rats was suggested to be through STAT3 activation [65]. 

During the discovery of adropin, its physiological role was linked to glucose homeostasis. It is thus important to understand the potential role of adropin in controlling hyperglycemia and its effect on insulin-sensitive tissues. 

In skeletal muscle, a study that was performed by Gao et al. showed that adropin played a crucial role in modulating glucose utilization in DIO mice with insulin resistance [20]. Adropin was able to promote glucose oxidation and diminish fatty acid oxidation in skeletal muscle, and that led to an increase in glucose uptake and enhanced mitochondrial function. The metabolic actions for enhancing mitochondrial function were mediated by suppressing the activity of peroxisome proliferator-activated receptor gamma coactivator-1a (PGC-1a), a transcription co-activator that regulates the expression of the genes that are involved in fatty acid oxidation. Moreover, adropin promoted skeletal muscle sensitization to insulin signaling actions by increasing insulin-induced Akt phosphorylation and the cell surface expression of glucose transporter 4 (GLUT4) [20].

In contrast, a study utilizing insulin-resistant hepatocytes showed that adropin could reduce glucose production in the liver. Adropin treatment downregulated the transcription of hepatic gluconeogenesis genes by inhibiting the binding site of transcription factors forkhead box protein O1(FoxO1) and cAMP-response element binding protein (CREB), along with their co-activators, peroxisome proliferator-activated receptor gamma coactivator 1-alpha (PGC1α) and CREB regulated transcription coactivator 2 (CRTC2), respectively, to the promoter of gluconeogenesis genes [47,66]. FoxO1-PGC1α and CREB-CRTC2 complex promoter binding is required to activate the transcription of genes that are involved in hepatic glucose production [67,68]. 

Interestingly, this effect of adropin was not observed in insulin-sensitive hepatocytes [66]. This research team earlier reported that a downregulation in adropin expression led to systemic insulin resistance in mice that were introduced to a high-fat diet for a long period [16]. 

Furthermore, it was reported that adropin was associated with incretins in obese males with type 2 diabetes receiving a 3-month treatment of liraglutide [69]. There was an increase in plasma adropin levels in those subjects. Liraglutide is an anti-diabetic agent, a specific glucagon-like peptide-1 receptor (GLP-1) agonist, also known as incretin mimetic molecule. Incretins are endogenous peptide hormones that are secreted by the GI tract to stimulate insulin secretion from pancreatic β-cells after meals [70,71,72,73]. 

It is worth mentioning that irisin, which is a peptide hormone involved in glucose homeostasis [5,74], has a similar effect on incretins, specifically GLP-1. Both adropin and irisin can enhance glucose-stimulated insulin secretion [75]. In addition, the peptide apelin increased plasma GLP-1 levels in rats that were intraperitoneally injected with apelin-13 [76]. However, the role of adropin in inducing incretin secretion and augmenting incretin effect remains unclear and needs more study to elucidate the mechanism by which it regulates these GI tract hormones Table 1.

### 4.2. Obesity

Obesity is a major health problem worldwide [44,81]. Studies performed on humans and animal models suggest that adropin may play a role in lipid metabolism and obesity.

C57BL/6J mice that were fed a high-fat diet exhibited a rapid increase in *Enho* gene expression, while fasting reduced the expression of this gene, when compared to the control mice. However, liver *Enho* gene expression declined when a high-fat diet was introduced to the mice for a longer period of time, suggesting a regulatory role of the *Enho* gene in nutrition, but the expression of adropin is diet-dependent [14]. Moreover, Kumar et al. generated an adropin knockout mice, which exhibited increased adiposity [82].

In humans, adropin level was negatively correlated with body mass index (BMI) [83] in diabetic patients, as overweight and obese patients had considerably reduced levels of adropin, compared to lean patients [57]. Moreover, when adropin levels were measured in plasma samples that were obtained from healthy subjects, it was found that the peptide levels correlated negatively with BMI and aging [84]. This observation was supported by studying patients who underwent bariatric surgery and monitoring serum adropin levels before and after surgery [85]. Serum adropin levels were higher 6 months after bariatric surgery than at baseline, leading to the conclusion that, in some patients, body mass reduction may restore the impaired production of adropin. Another study was conducted to investigate the role of adropin in children with obesity or metabolic syndrome [86]. The results of this study showed that—there was no significant difference between the plasma level of adropin in obese children, and those individuals with normal weight Table 1.

### 4.3. Cardiovascular Diseases

There is overwhelming evidence that cardiovascular diseases are common in patients suffering from diabetes mellitus [77,87,88,89,90,91,92]. Several studies and reports indicate the involvement of adropin in the functioning of the cardiovascular system. As mentioned previously, the immunoreactivity of adropin has been detected in many tissues, including the three layers of the heart [24].

High cardiac fatty acid oxidation rates and impaired cardiac insulin signaling are associated with decreased cardiac efficiency and various cardiac diseases [93]. Altamimi et al. investigated the effect of adropin on cardiac energy metabolism, insulin signaling and cardiac efficiency [94]. C57Bl/6 mice were injected with a secretable form of adropin (450 nmol/kg, i.p.) three times over 24 h, then they were fasted, and the hearts were isolated and perfused. Altamimi et al. demonstrated that adropin administration improved cardiac function, cardiac efficiency and coronary flow, compared to the untreated mice. Moreover, by measuring glucose and palmitate contribution in catabolic pathways for ATP production, the important role of adropin on the preference of cardiac glucose oxidation and the inhibition of cardiac fatty acid oxidation were reported. In cardiomyocytes, adropin regulates cell bioenergetics through GPR19 activation. The receptor activation leads to stimulation of p44/42 phosphorylation and, consequently, the downregulation of pyruvate dehydrogenase kinase 4 (PDK4) and pyruvate dehydrogenase (PDH) phosphorylation [95,96,97,98].

Hyperlipidemia is a risk factor that is associated with cardiovascular diseases [99]. Akcilar et al. demonstrated the role of a low dose of adropin in reducing hyperlipidemia in rats that were fed a high-fat diet. A reduction in the levels of serum triglycerides, total cholesterol and low-density lipoprotein cholesterol (LDL-C), as well as an increment in the level of high-density lipoprotein cholesterol (HDL-C) were reported [100]. Additionally, adropin administration reduced the mRNA expression of pro-inflammatory cytokines, tumor necrosis factor alpha (TNF-α) and interleukin-6 (IL-6). This suggests that adropin may have an anti-inflammatory role in the liver and possibly in other organs, including the heart. 

Interestingly, a recent meta-analysis found an association between serum adropin and coronary artery disease (CAD) [101]. The study stated that the serum adropin level in patients with CAD was lower than in healthy individuals, indicating that the decrease in adropin concentration might play an important role in the development of CAD. Another cardiovascular disease, which has been correlated with adropin, is atrial fibrillation. Atrial fibrillation is a condition of abnormal heart rhythm [102,103]. Decreased serum adropin concentrations were found in atrial fibrillation patients compared with healthy controls. Patients with chronic atrial fibrillation had a significantly reduced serum adropin concentration compared with control patients. Hence, adropin deficiency may contribute to the development and progression of atrial fibrillation [104] Table 1.

### 4.4. Inflammation

Researchers and scientists have also investigated the role of adropin in inflammation and related diseases such as atherosclerosis. Atherosclerosis is a chronic inflammatory disease in response to injury of the arterial wall and the formation of plaque [80]. Vascular inflammation stimulates the expression of certain adhesion molecules, such as intercellular adhesion molecule 1, vascular cell adhesion molecule 1 in endothelial cells. These adhesion molecules stimulate monocyte adhesion to endothelial cells and monocyte infiltration into the subendothelial space, causing an accumulation of macrophage foam cells. Chronic inflammation of the cardiovascular system is a common feature of chronic diseases including diabetes mellitus, hyperlipidemia and nosocomial conditions [105,106,107]. Hormones such as adropin and plant-based antioxidants have been used to mitigate the adverse effects of these vascular lesions. Adropin is expressed in human endothelial cells [23], and it has been shown earlier that adropin inhibits tumor necrosis factor α (TNFα). Sato et al. investigated the relationship between TNFα, monocyte adhesion, human endothelial cells, atherosclerosis and adropin. The experiment involved incubating human endothelial cells with adropin and TNFα and assessing the expression of the adhesion molecules that are involved in atherosclerosis. The results showed that an incubation of adropin alone had no significant effect on the mRNA expression of these adhesion molecules, which are usually stimulated by TNFα. However, when adropin and TNFα were both incubated with human endothelial cells, adropin suppressed the TNFα-induced mRNA expression of adhesion molecules, suggesting a role for adropin in the anti-atherosclerosis process by inhibiting endothelial cells’ adhesion molecules via the suppression of TNFα [108].

In the case of obesity, the infiltration of macrophages into adipose tissues causes chronic inflammation. Adipocytes secrete cytokines such as TNFα and MCP-1 that attract macrophages and regulatory T cells, leading to fat inflammation. Adropin regulates the expression of PPAR-γ by activating the AKT pathway, thus inhibiting the differentiation of 3T3-L1 preadipocytes into mature adipocytes and consequently reducing fat accumulation and fat inflammation [109]. 

In another study, lower adropin plasma levels and increased inflammation markers such as TNFα and interleukin-6 (IL-6) were reported in male patients with moderate and severe obstructive sleep apnea, compared to healthy individuals [110].

Furthermore, in order to investigate how adropin could affect hepatocyte inflammation and injury in nonalcoholic steatohepatitis (NASH), immunohistochemistry using the inflammation markers F4/80, CD45 and MCP-1, and a gene expression analysis for TNFα and IL-6 genes, were performed using liver tissues from adropin knockout C57BL/6J mice and the control wild-type, which were fed a methionine-choline deficient diet [111]. Methionine-choline deficient diet is the classic dietary model for studying NASH, and usually, rodents consuming this diet develop steatohepatitis, necroinflammation, and fibrosis, similar to human NASH [78]. The pathohistological analysis showed a higher signal of F4/80, CD45 and MCP1 and a substantial induction of genes TNFα and IL-6 in adropin knockout mice. These results indicate the presence of elevated inflammatory responses in adropin knockout mice, when compared to that of the wild-type mice [111] (Table 1).

### 4.5. Cell Proliferation and Differentiation

Far beyond the classical action, adropin can stimulate cell proliferation and differentiation. Lovren et al. showed that adropin has the ability to induce the proliferation and capillary-like tube formation of endothelial cells that stimulate angiogenesis [23]. Adropin upregulated endothelial NO synthase expression through VEGFR2 2-PI3K-Akt and VEGFR2-extracellular signal-regulated kinase pathways to reduce inflammation.

Furthermore, in rat primary preadipocytes and 3T3-L1 cells, preadipocyte proliferation was increased by adropin treatment, while the differentiation of those preadipocytes into mature adipocytes was reduced [79]. The suppression of adipogenic markers and lipid accumulation demonstrate the important role of adropin in the fight against obesity [109]. The same research team reported a similar effect of adropin on primary brown preadipocytes that were isolated from the interscapular region in rat [112]. 

**Figure 2:**
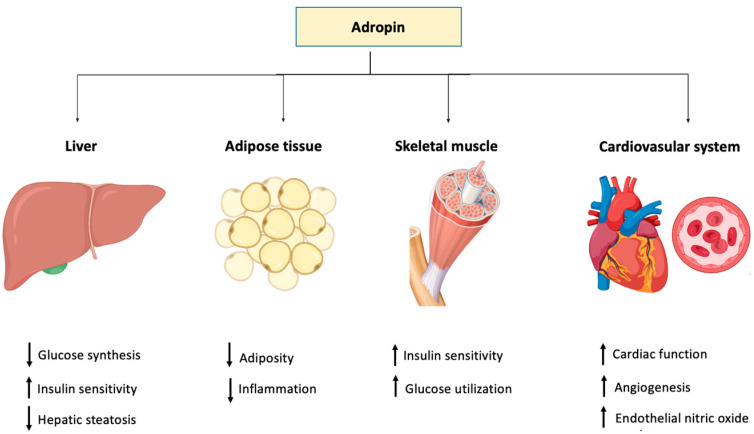
Graphical representation of the function of adropin in different body tissues.

On the other hand, it was reported that adropin downregulated the proliferation and migration of human aortic smooth muscle cells (HASMCs) in vitro, providing evidence that the peptide is also protective against atherosclerosis [108] (Table 1). The role of adropin in different tissues and organ systems is depicted in Figure 2.

## 5. Characteristics of Adropin Knockout Mice

Knockout mice are usually used to study what happens in an organism when a particular gene is absent. Studying knockout mice can provide information about how the knocked-out gene normally functions, including the gene’s biochemical, developmental, physical and behavioral roles.

It was shown that adropin knockout C57BL/6J mice exhibited remarkable insulin resistance, dyslipidemia, failure in the suppression of endogenous glucose production in a hyperinsulinemic condition and increased adiposity in liver [82]. These knockouts were generated by targeting the open reading frame of the *Enho* gene—specifically in exon 2—and carried out Cre-mediated deletion using Cre-LoxP recombination technology [82].

In another study, adropin knockout C57BL/6J mice were used to investigate the pathogenicity of fatty pancreas [58]. Interestingly, these mice developed fatty pancreas and showed a significant decrease in the amount of regulatory T cells; regulatory T cells are involved in controlling the inflammatory state [113]. Other pathological conditions included an increased severity of obesity-related impaired glucose homeostasis, lipid metabolism disorder and reduced endothelial nitric oxide synthase phosphorylation, as reported in earlier studies. Nitric oxide synthase is an important molecule regulating endothelial function including blood flow and vascular integrity [114,115].

Chen et al., as well as Gao et al., used clustered regularly interspaced short palindromic repeats (CRISPR/Cas9) technology and *Enho* single-guide RNA, the latest tool in genome editing, to generate adropin knockout C57BL/6J mice [34,58]. They reported a higher susceptibility to developing myeloperoxidase and anti-neutrophil cytoplasm autoantibody (MPO-ANCA)-associated lung injury in adropin knockout C57BL/6J mice [34].

## 6. Conclusions

In summary, adropin, a 76-amino acid peptide, is membrane-bound and secreted by cells. It is present in the endothelial cells of the capillaries of the brain, liver and kidney. Adropin is involved in several biological activities, and it is regulated by nutrients including lipids and carbohydrates. The administration of adropin can enhance the oxidation of glucose, with a concomitant reduction in fatty acid oxidation in skeletal muscle cells. Adropin has a protective effect in type 2 diabetes mellitus, as it can reduce insulin resistance and prevent the development of obesity by enhancing lipid catabolism. It promotes insulin signaling pathways through Akt phosphorylation and the cell surface expression of GLUT4. Additionally, adropin modulates lipid metabolism by regulating the expression of hepatic lipogenic genes and the PPARγ receptor, the major regulator of lipogenesis. Therefore, adropin-based treatments could emerge as a new line of therapy against glucose and lipid metabolism-related diseases.

It has also been shown that adropin improves coronary blood flow and cardiac function. Adropin promotes cardiac glucose oxidation and the inhibition of cardiac fatty acid oxidation, enhancing cardiac energy metabolism and cardiac efficiency. Moreover, the anti-inflammatory effect of adropin can suppress TNFα expression in atherosclerosis.

Studies showed that *Enho* gene expression, adropin serum/plasma levels and/or protein expression level in tissue can fluctuate, according to the type of illness. These conditions include but are not limited to diabetes mellitus, obesity, cardiovascular diseases and inflammation. Thus, adropin could also be used as a diagnostic biomarker to detect these clinical conditions, especially metabolic and cardiovascular diseases. However, more research needs to be carried out to understand its mechanism of action in specific tissues and to discover if it acts on a specific receptor, other than GPR19.

## Figures and Tables

**Table 1:** Effect of adropin on metabolic parameters.

Organ/Systems/Condition	Effect	Reference
Liver	Increases the expression of hepatic lipogenic genes and PPARγ	[14]
Cardiovascular system	Increases angiogenesis, blood flow, capillary density, and protects endothelial cells	[23]
	Improves cardiac function and coronary flow	[77]
Diabetes mellitus	Low adropin level increases the risk of chronic complications of diabetes	[63,64,65]
	Stimulates insulin signal pathways by promoting Akt phosphorylation	[20]
Fat	Suppresses lipid accumulation	[78,79]
Inflammation	Inhibits TNF-α	[80]
